# Plasma endostatin correlates with hypoxia and mortality in COVID-19-associated acute respiratory failure

**DOI:** 10.2217/bmm-2021-0111

**Published:** 2021-10-20

**Authors:** Sana Asif, Thoralph Ruge, Anders Larsson, Sara Bülow Anderberg, Miklos Lipcsey, Robert Fritiof, Michael Hultström

**Affiliations:** ^1^Department of Surgical Sciences, Anaesthesiology & Intensive Care Medicine, Uppsala University, Uppsala, 751 85, Sweden; ^2^Department of Clinical Sciences, Lund University, Malmö, Sweden, Lund, 221 00, Sweden; ^3^Department of Medical Sciences, Uppsala University, Uppsala, 751 85, Sweden; ^4^Department of Surgical Sciences, Hedenstierna Laboratory, Uppsala University, Uppsala, 751 85 Sweden; ^5^Department of Medical Cell Biology, Unit for Integrative Physiology, Uppsala University, Uppsala, 751 85, Sweden

**Keywords:** ARDS, COVID-19, endostatin, hypoxia, inflammation, intensive care, pulmonary injury

## Abstract

**Background:** The contribution of endothelial injury in the pathogenesis of COVID-19-associated acute respiratory distress syndrome (ARDS) and resulting respiratory failure remains unclear. Plasma endostatin, an endogenous inhibitor of angiogenesis and endothelial dysfunction is upregulated during hypoxia, inflammation and progress of pulmonary disease. **Aim:** To investigate if plasma endostatin is associated to hypoxia, inflammation and 30-day mortality in patients with severe COVID-19 infection. **Method:** Samples for blood analysis and plasma endostatin quantification were collected from adult patients with ongoing COVID-19 (n = 109) on admission to intensive care unit (day 1). Demographic characteristics and 30-day mortality data were extracted from medical records. The ability of endostatin to predict mortality was analyzed using receiving operating characteristics and Kaplan–Meier analysis with a cutoff at 46.2 ng/ml was used to analyze the association to survival. **Results:** Plasma endostatin levels correlated with; PaO_2_/FiO2 (r = -0.3, p < 0.001), arterial oxygen tension (r = -0.2, p = 0.01), lactate (r = 0.2, p = 0.04), C-reactive protein (r = 0.2, p = 0.04), ferritin (r = 0.2, p = 0.09), D-dimer (r = 0.2, p = 0.08) and IL-6 (r = 0.4, p < 0.001). Nonsurvivors at 30 days had higher plasma endostatin levels than survivors (72 ± 26 vs 56 ± 16 ng/ml, p = 0.01). Receiving operating characteristic curve (area under the curve 0.7) showed that plasma endostatin >46.2 ng/ml predicts mortality with a sensitivity of 92% and specificity of 71%. In patients with plasma endostatin >46.2 ng/ml probability of survival was lower (p = 0.02) in comparison to those with endostatin <46.2 ng/ml. **Conclusion:** Our results suggest that plasma endostatin is an early biomarker for disease severity in COVID-19.

## Background

Respiratory failure caused by acute respiratory distress syndrome (ARDS) is the most common manifestation of COVID-19, requiring admission to the intensive care unit (ICU) and has a fatality rate of approximately 20–50% [1–4]. Pathophysiologically, COVID-19-induced ARDS is characterized by acute inflammatory damage to the alveolar capillary barrier resulting in increased vascular permeability, endothelial injury and poor gas exchange causing hypoxia. Depending on infection severity, gaseous exchange may become severely impaired, warranting ICU admission and mechanical ventilation [5–7]. The median onset of COVID-19-induced respiratory failure is reported to be between 8–12 days. [5,6].

Endostatin, a 20 kDa C-type fragment of collagen XVIII, is one of the most potent endogenously produced inhibitors of angiogenesis. It prevents endothelial cell migration, proliferation and induces endothelial cell damage by interfering with vascular endothelial growth factor (VEGF) signaling [8–10]. Elevated plasma endostatin levels in humans are reported to be involved in the pathogenesis of tumors, chronic inflammatory conditions, atherosclerosis, chronic kidney failure and cardiovascular injury [11–13]. Additionally, endostatin was reported to be associated with adverse outcome and morality in patients presenting at the emergency department with acute dyspnoea [13,14].

The aim of the present study was to investigate if plasma endostatin levels were elevated in patients admitted to intensive care with severe COVID-19 and to determine if endostatin levels correlate to pulmonary hypoxia, inflammation and short-term mortality in these patients.

## Methods & materials

The present study is a prospective observational study carried out at a combined medical and surgical intensive care unit at Uppsala University hospital, a tertiary care hospital in Sweden. The study was approved by the National Ethical Review Agency (EPM; no. 2020–01623). Informed consent was obtained from the patient, or next of kin if the patient was unable to give consent. Less than 10% of the patients declined participation. There was no exclusion criteria. The Declaration of Helsinki and its subsequent revisions were followed. The protocol of the study was registered *a priori* (Clinical Trials ID: NCT04316884). STROBE guidelines were followed for reporting.

### Patients

Adult (18 years or older) patients admitted to the intensive care unit with an ongoing COVID-19 between March and August 2020, with informed consent, and analyzed levels of endostatin in plasma were included in this study. COVID-19 was diagnosed with a positive reverse transcriptase PCR on nasopharyngeal swabs, conducted at the Uppsala University hospital laboratory.

### Baseline characteristics

Baseline parameters such as age, sex and BMI were recorded on admission to the ICU. Smoking habits (yes/not any longer vs no) and information on associated comorbidities, such as pulmonary disease, hypertension, diabetes mellitus, were extracted from the patients’ medical records. Subsequently, data on 30 day mortality post ICU admission, ventilation modes (invasive versus non-invasive), duration of illness on hospital admission (days) and medical treatment were also collected from medical records of each patient.

### Clinical parameters & biochemistry

Simplified acute physiology score 3, sequential organ failure assessment score, lung function assessed by PaO_2_/FiO_2_ ratio, pH, PaO^2^, PaCO_2_, as well as blood lactates on arterial blood gas were recorded on admission to ICU, designated as day 1. The PaO2/FiO2 ratio obtained on day of ICU admission was used to categorize hypoxemia into mild (PaO^2^/FiO^2^ 200–300 mmHg), moderate (PaO_2_/FiO_2_ 100–200 mmHg) and severe (PaO_2_/FiO_2_ <100 mmHg) as per Berlin ARDS classification. Furthermore, blood samples were also collected on day 1 of ICU admission for full blood count, C-reactive protein (CRP), IL-6, ferritin and D-dimer. Full blood count was analyzed on a Sysmex XN™ instrument (Sysmex, Kobe, Japan) while CRP, ferritin, D-dimer and IL-6 were quantified on an Architect ci16200 (Abbott Laboratories, IL, USA). On admission to ICU, patients with acute kidney injury were identified according to Kidney Disease: Improving Global Outcome Criteria (KDIGO).

### Endostatin

Plasma samples for endostatin quantification were collected on day 1 of ICU admission. Endostatin in plasma was measured using a sandwich ELISA kit for human endostatin (DY1098, R&D Systems, MN, USA) according to manufacturer’s instructions.

Briefly, the microtiter plates were coated with a monoclonal anti-endostatin antibody. After washing, the plates were blocked with 1% bovine serum albumin in 0.02 M NaH_2_PO^4^, 0.15 M NaCl and pH 7.2 bovine serum albumin (BSA-buffer). After a washing step, the samples were added diluted 1:100 in BSA-buffer together with calibrators. The eight calibration points were analyzed as duplicates and the samples were analyzed as singletons. After incubation and washing, a biotinylated anti-endostatin antibody diluted in BSA-buffer was added. After further incubation and washing cycle, the streptavidin–HRP conjugate was added. The plate was incubated and then washed, and a substrate solution was added. The substrate development was stopped, and the color intensity was measured. Values are expressed as ng/ml. The assays had a total coefficient of variation of approximately 6% and the limit of detection was 6.25 ng/ml. All laboratory tests were performed blinded without any knowledge of patient outcome.

### Statistics

Continuous variables are presented as median (interquartile range) or mean ± standard deviation as appropriate. Data plotting and statistical analysis were performed using Prism version 6 for Macintosh software (Graphpad, CA, USA). Mann–Whitney U test was used for group comparisons and Spearman Rank to assess correlation between variables. Sensitivity and specificity were calculated at the best cutoff for plasma endostatin for 30-day mortality in the receiver operating characteristic (ROC) curve to calculate and assess the predictive accuracy. Kaplan–Meier survival estimate was used to analyze the probability survival using the plasma endostatin cutoff. A p-value <0.05 was considered significant.

## Results

The baseline characteristics of patients are illustrated in Table 1. In our cohort, 76% of COVID-19 patients were males, with a mean age of 61 years and a BMI of 29 (kg/m^2^). History of previous or active smoking was recorded in 24% of the patients. The most common comorbidities were hypertension (58%), pulmonary disease (28%) and diabetes mellitus (29%). The median time from symptom onset to hospital admission was between 10 and 34 days. In our cohort, 92% of patients (n = 100) required either high-frequency oscillatory ventilation (HFNOV) or noninvasive ventilation support before being admitted to the ICU. On the day of ICU admission, all of the COVID-19 patients (n = 109), had an PaO_2_/FiO_2_ ratio of <300 mmHg (150 ± 67 mmHg). Out of total, 56% (n = 61) were intubated and 44% (n = 48) required HFNOV at a flow rate greater than 60 l/min. Ventilation modes, however, did not affect short-term mortality; 30% (n = 18) of intubated patients and 19% (n = 9) of nonintubated died in 30 days of ICU admission (p = 0.2). During the course of ICU stay, all patients included in this study received dalteparin 100–200 mg/kg once daily as medical treatment. Out of total 109, 68 patients (56%) developed acute kidney injury due to increased creatinine or loss of urine production.

**Table 1.** Background and associated comorbidities and clinical parameters of COVID-19 patients (n = 109), reported as median and interquartile range or n (%).

Variables	COVID-19 (n = 109) n (%), median (IQR)
Age (years)	61 (24–86)
Male, n (%)	83 (76)
BMI (kg/m^2^)	29 (19–51)
Previous or active smoking, n (%)	24 (22)
SAPS3	53 (32–88)
SOFA score on admission	6 (0–12)
Duration of illness on admission (days)	10 (5–34)
**Co-morbidities, n (%)**	
Pulmonary disease	28 (26)
Hypertension	58 (53)
Diabetes mellitus	29 (27)

IQR: Interquartile range; SAPS3: Simplified acute physiology score; SOFA: Sequential organ failure assessment.

**Figure 1.**
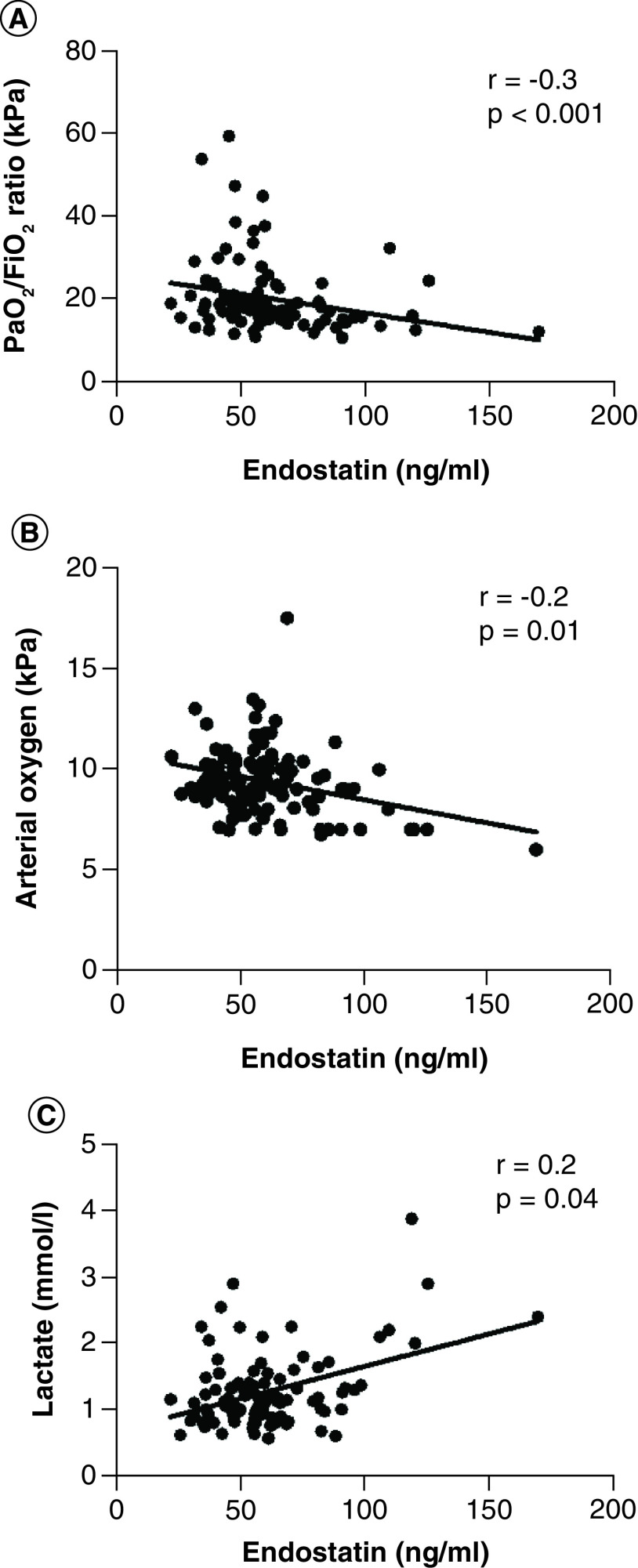
Association of plasma endostatin to hypoxia. Relationship of plasma endostatin to **(A)** poor gas exchange as measured as low PO_2_/FiO_2_ ratio (r = -0.3, p < 0.001), **(B)** hypoxia measured as low arterial oxygenation tension (r = -0.2, p = 0.01) and **(C)** anaerobic metabolism measured by blood lactate (r = 0.2, p = 0.04), in COVID-19 patients (n = 109) admitted to the ICU. Each point on the graph represents individual values, collected on day 1 of ICU admission, solid line indicates linear regression, (r) is Spearman rank correlation coefficient and p is correlation’s significance level. The graph illustrates a strong correlation of plasma endostatin to lung function and arterial hypoxia in COVID-19 patients. ICU: Intensive care unit.

**Figure 2.**
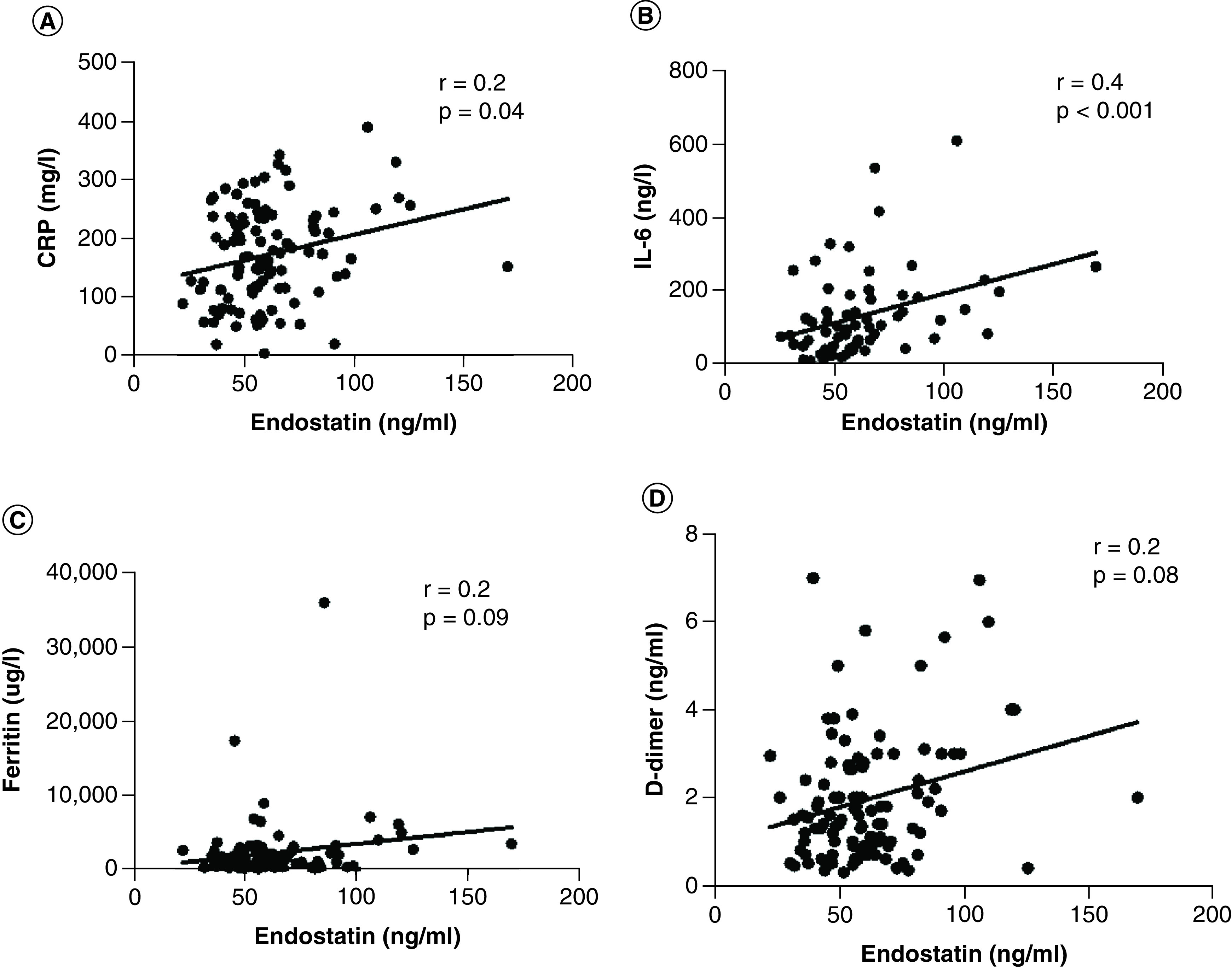
Association of plasma endostatin to inflammation. Correlation between plasma endostatin and inflammatory biomarkers by **(A)** CRP (r = 0.2, p = 0.04), **(B)** IL-6 (r = 0.4, p < 0.001), **(C)** ferritin (r = 0.2, p = 0.09) and **(D)** D-dimer (r = 0.2, p = 0.08) in COVID-19 patients (n = 109) during intensive care. Each point on the graph represents individual values collected on day 1 of ICU admission, whereas the solid line represents the linear regression, r is Spearman rank correlation coefficient and p is correlation’s significance level. The graph shows significant correlation between plasma endostatin and inflammatory markers such as CRP and IL-6. No significant association was observed between plasma endostatin and ferritin or D-dimer. CRP: C-reactive protein.

On day 1 of ICU admission, the average arterial oxygen saturation measured in our cohort of severe COVID-19 (n = 109), was (94 [79–98]%), PO_2_/FiO_2_ ratio was (150 ± 67 mmHg/20 ± 9 kPa), pH (7.4 ± 0.0), arterial PO_2_ (9.3 ± 1.7 kPa), arterial PCO_2_ (4.7 ± 0.9 kPa), lactate (1.2 ± 0.5 mmol/l), CRP (173 ± 82 mg/l) ferritin (2234 ± 4190 μg/l), IL-6 (135 ± 117 ng/l) and D-dimer (2.4 ± 4.0 ng/ml). Moreover, plasma endostatin levels were found to be correlated to poor gas exchange as measured by PaO_2_/FiO_2_ ratio (r = -0.3, p < 0.001); hypoxia as illustrated by arterial oxygen tension (r = -0.2, p = 0.01); and to anaerobic metabolism assessed by blood lactate (r = 0.2, p = 0.04; Figure 1, Table 2). Similarly, at day 1 higher levels of acute phase proteins and plasma cytokines were observed in patients with acute COVID-19. Moreover, plasma endostatin levels were found to be correlated to levels of acute phase proteins and inflammatory cytokines such as CRP (r = 0.2, p = 0.04), ferritin (r = 0.2, p = 0.09), D-dimer (r = 0.2, p = 0.08) and IL-6 (r = 0.4, p < 0.001; Figure 2, Table 2). The number of patients who did survive up to 30 days were (n = 87, 80%) in comparison to those who died in this period (n = 22, 20%). Of those who survived (n = 79, 91%) were discharged from the ICU within 30 days in comparison to (n = 8, 9%) who were still admitted and required ventilatory support.

**Figure 3.**
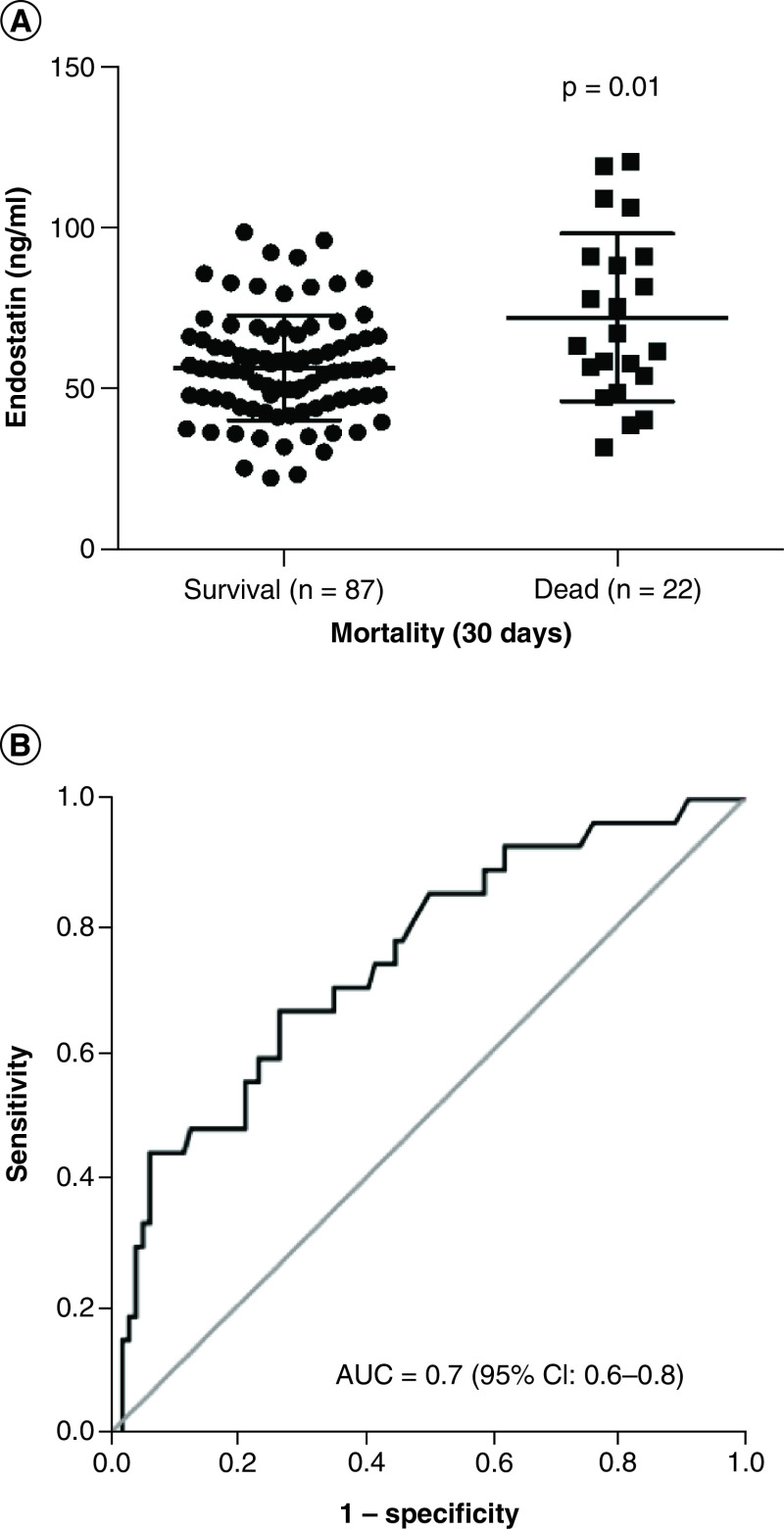
Plasma endostatin and its effects on short term mortality in COVID-19. **(A)** On day 1 of ICU admission, plasma endostatin concentration in patients critically ill with COVID-19 was higher in non-survivors (n = 22) in comparison to those who survived (n = 87) to 30 days, (72 ± 26 vs 56 ± 16 ng/ml; p = 0.01. Significance (p < 0.05) was determined by Mann–Whitney test. **(B)** Receiver operating characteristic curve of plasma endostatin with a cutoff at 46.2 ng/ml yields an AUC of 0.70 (p = 0.01) for predicting 30-days mortality in COVID-19 patients (n = 109). AUC: Area under curve; ICU: Intensive care unit.

**Figure 4.**
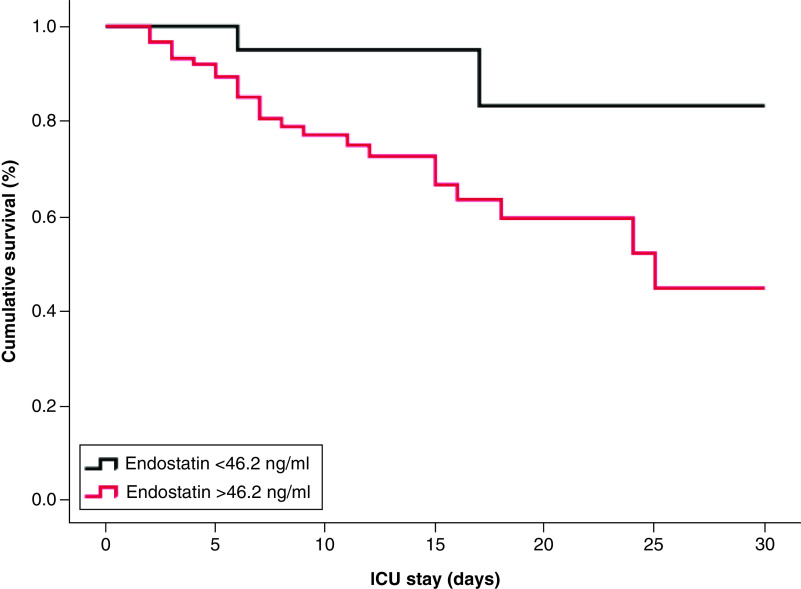
Higher levels of plasma endostatin (>46.2 ng/ml) are associated with poor survival (hazard ratio: 4.8; 95% CI: 1.07–22; p = 0.02) by Kaplan–Meier estimation in n = 109 critical COVID-19 patients after intensive care unit admission.

Finally, plasma endostatin was higher in patients who did not survive to 30 days (72 ± 26 vs 56 ± 16 ng/ml; p = 0.01; Figure 3A). Plasma endostatin alone yielded an area under the curve of 0.7 (95% CI: 0.6–0.8; Figure 3B). The cut-off value for plasma endostatin generated from the receiving operating characteristic (46.2 ng/m) predicts COVID-19-associated short-term mortality with a sensitivity of 92% and specificity of 71%. Plasma endostatin levels >46.2 ng/ml are associated with a significantly increased risk of death (hazard ratio [HR] 4.8; 95% CI: 1.07–22; p = 0.02; Figure 4).

## Discussion

The main result of the present study is that plasma endostatin is associated with hypoxia, inflammation and associated to short-term mortality in intensive care patients with COVID-19. To our knowledge, this is the first study investigating endostatin in critically ill COVID-19 patients. There are several potential explanations for the identified correlations.

### Endostatin in hypoxia

Hypoxia in COVID-19, is driven by altered pulmonary gas exchange, inflammation, dysregulated immune response and pulmonary endothelial injury [15–18]. Release of endostatin has been shown to increase in hypoxic milieus such as during exercise and cycling.

### Balance between VEGF & endostatin

Survival of pulmonary endothelial cells rely on a balance between VEGF and endostatin [10,19]. In patients with ongoing pulmonary injury, the angiogenic balance may be switched to an anti-angiogenic milieu facilitating endostatin release. This endostatin participates in negative feedback regulation of the activity of VEGF and a decrease in ratio of VEGF to endostatin. This results in a loss of pulmonary endothelial cells, apoptosis and cell death [10]. For instance, in chronic obstructive pulmonary disease, plasma endostatin was significantly increased in patients with, and strongly associated with disease severity, decreased lung function, increased systemic inflammation and increased number of exacerbations [20,21]. Additionally [22], patients with pulmonary arterial hypertension have high levels of circulating endostatin [22,23], which correlates to worsened patient outcome.

**Table 2.** Spearman Rank correlation coefficients (r) and corresponding p-values for the observed correlation between plasma endostatin^†^ and levels of hypoxic and inflammatory markers in COVID-19 (n = 109) on admission to intensive care unit.

Variables	Mean ± SD, median (IQR)	r^‡^	p-value^§^	95% CI
PO_2_/FiO_2_ ratio (kPa/mmHg)	20 ± 9/150 ± 67	-0.3	<0.001	-0.5–0.2
Arterial oxygen tension (kPa)	9.3 ± 1.7	-0.2	0.01	-0.4–0.03
Lactate (mmol/l)	1.2 ± 0.5	0.2	0.04	-0.0–0.4
CRP (mg/l)	173 ± 82	0.2	0.04	0.0–0.4
IL-6 (ng/l)	135 ± 117	0.4	<0.001	0.2–0.6
Ferritin (μg/l)	2234 ± 4190	0.2	0.09	-0.0–0.4
D-dimer (ng/ml)	2.4 ± 4.0	0.2	0.08	-0.03–0.3

† Plasma endostatin (mean ± SD, 61 ± 23 ng/ml).

‡ Spearman Rank correlation coefficients (r).

§ p-values calculated by *t*-test at 95%CI.

CRP: C-reactive protein; IQR: Interquartile range; SD: Standard deviation.

### Endostatin in inflammation

Inflammation is well known to stimulate extracellular matrix turnover and collagen deposition, which is associated with endostatin release as it is a collagen fragment. The increase of plasma endostatin has also been linked to an increase of both systemic and local inflammation, where it is thought to be tied to increased collagen turnover rather than the regulation of angiogenesis [23].

Taken together, profound hypoxia as well as dysregulated inflammation, as observed in COVID-19 patients, may trigger the release of endostatin in the lungs. These two conditions shift the angiogenic switch from a proangiogenic milieu to an antiangiogenic environment with corresponding increased levels of endostatin possibly mirroring endothelial cell dysfunction, autophagy and pulmonary injury.

Our investigation has several strengths, including being a prospective cohort with comprehensive clinical characterization. Plasma samples were collected at admission, which allows identifications and characterization of disease-associated risk factors. A limitation of the study is that as a single-center study with a relatively low number of included patients, nevertheless, as an ICU-based study the severe disease still gives a reasonable number of events for statistical analysis. Furthermore, the criteria for ICU admission was respiratory failure where HFNOV at a flow rate of 60 l/min was insufficient, which makes it difficult to apply outside the ICU settings. Further studies are warranted.

## Conclusion

In conclusion, plasma endostatin is associated with hypoxia and inflammation in COVID-19 and admission plasma concentration >46.2 ng/ml predicts out come. This particular mediator may be a useful marker for disease severity in COVID-19.

**Summary points**

**Background**

- Endostatin, an endogenously produced inhibitor of angiogenesis, is a marker of endothelial injury.
- Elevated endostatin levels are reported to be involved in the pathogenesis of various tumors and chronic inflammatory conditions.

**Endostatin in COVID-19**

- COVID-19-associated acute respiratory distress syndrome, characterized by hypoxia, inflammation endothelial injury and subsequent respiratory failure is a common presentation of COVID-19.
- The contribution of endostatin in COVID-19-induced acute respiratory distress syndrome remains unclear.
- Thereby in this prospective observational study, we investigated if plasma endostatin is associated to hypoxia, inflammation and 30 days mortality in patients admitted to the intensive care unit with severe COVID-19.
- A strong correlation was found between plasma endostatin and markers of hypoxia, and inflammation.
- Moreover, elevated plasma endostatin levels were associated with higher mortality. At a cut-off value of 46.2 ng/ml plasma endostatin predicts mortality with a sensitivity of 92% and specificity of 71%.

**Conclusion & future perspectives**

- Plasma endostatin is an early biomarker of disease severity and poor outcome in COVID-19.
- Multicenter studies with serial follow up of plasma endostatin levels are warranted.
